# Effects of a soft robotic exosuit on the quality and speed of overground walking depends on walking ability after stroke

**DOI:** 10.1186/s12984-023-01231-7

**Published:** 2023-09-01

**Authors:** Lizeth H. Sloot, Lauren M. Baker, Jaehyun Bae, Franchino Porciuncula, Blandine F. Clément, Christopher Siviy, Richard W. Nuckols, Teresa Baker, Regina Sloutsky, Dabin K. Choe, Kathleen O’Donnell, Terry D. Ellis, Louis N. Awad, Conor J. Walsh

**Affiliations:** 1 Harvard John A. Paulson School of Engineering and Applied Sciences, Cambridge, MA USA; 2 Wyss Institute for Biologically Inspired Engineering, Boston, MA USA; 3 ZITI Institute of Computer Engineering, Heidelberg University, Heidelberg, Germany; 4 Institute for Biomedical Engineering, ETH Zürich, Zürich, Schweiz; 5 Department of Physical Therapy, Boston University, Boston, MA USA

**Keywords:** Soft exosuit, Exoskeleton, Stroke, Walking speed, Push-off, Ground clearance, Rehabilitation

## Abstract

**Background:**

Soft robotic exosuits can provide partial dorsiflexor and plantarflexor support in parallel with paretic muscles to improve poststroke walking capacity. Previous results indicate that baseline walking ability may impact a user’s ability to leverage the exosuit assistance, while the effects on continuous walking, walking stability, and muscle slacking have not been evaluated. Here we evaluated the effects of a portable ankle exosuit during continuous comfortable overground walking in 19 individuals with chronic hemiparesis. We also compared two speed-based subgroups (threshold: 0.93 m/s) to address poststroke heterogeneity.

**Methods:**

We refined a previously developed portable lightweight soft exosuit to support continuous overground walking. We compared five minutes of continuous walking in a laboratory with the exosuit to walking without the exosuit in terms of ground clearance, foot landing and propulsion, as well as the energy cost of transport, walking stability and plantarflexor muscle slacking.

**Results:**

Exosuit assistance was associated with improvements in the targeted gait impairments: 22% increase in ground clearance during swing, 5° increase in foot-to-floor angle at initial contact, and 22% increase in the center-of-mass propulsion during push-off. The improvements in propulsion and foot landing contributed to a 6.7% (0.04 m/s) increase in walking speed (*R*^*2*^ = 0.82). This enhancement in gait function was achieved without deterioration in muscle effort, stability or cost of transport. Subgroup analyses revealed that all individuals profited from ground clearance support, but slower individuals leveraged plantarflexor assistance to improve propulsion by 35% to walk 13% faster, while faster individuals did not change either.

**Conclusions:**

The immediate restorative benefits of the exosuit presented here underline its promise for rehabilitative gait training in poststroke individuals.

**Supplementary Information:**

The online version contains supplementary material available at 10.1186/s12984-023-01231-7.

## Background

Stroke is a leading cause of serious long-term disability that results in a slow, unstable, and energetically inefficient gait. Paresis of the muscles on one side of the body contributes to asymmetric walking patterns poststroke. Impaired plantarflexor muscle activity on the paretic side results in reduced propulsive force [1], whereas impaired dorsiflexor activity results in reduced ground clearance and impaired limb loading [2–5]. Together, these impairments increase the risk of falling, which is often compensated for by hip hiking and hip circumduction strategies [6, 7]. These mobility deficits can hinder social participation and affect the quality of life [8], warranting the development of interventions that restore paretic plantarflexor and dorsiflexor function during walking [9].

For people with neurological conditions, wearable robots have the potential to help restore mobility. Rigid exoskeletons that provide full body weight and limb advancement support have been shown to be beneficial for non-ambulatory individuals with for instance a complete spinal cord injury [10, 11], but mixed results are found for ambulatory individuals with gait impairments such as most stroke survivors [10–13]. In fact, the high levels of assistance might reduce the user’s neuromuscular activity [14–16]. Because active engagement is crucial for the experience-dependent plasticity that underlies motor recovery, the partial support provided by soft robotic exosuits are a promising therapeutic alternative to keep neuromuscular slacking to a minimum.

We developed a soft robotic exosuit to provide paretic plantarflexor assistance to enhance propulsion during the push-off phase and paretic dorsiflexor assistance to improve ground clearance during the swing phase and foot landing during the loading phase [17, 18]. This lightweight wearable device applies assistance via Bowden cables that connect to garment-like, functional textile anchors on the shank and foot. The textile-based interface allows exosuits to operate in parallel with the user’s paretic muscles to augment, not replace, their movements. Our previous studies with a tethered exosuit and a preliminary version of the portable exosuit (5 kg total weight including motors and batteries worn at the waist) reported improvements in the mechanics, energetics, and functional walking capacity of a small cohort of community-dwelling people poststroke compared to walking with an exosuit unpowered [17, 19, 20] or walking without an exosuit [19, 21–23].

**Fig. 1:**
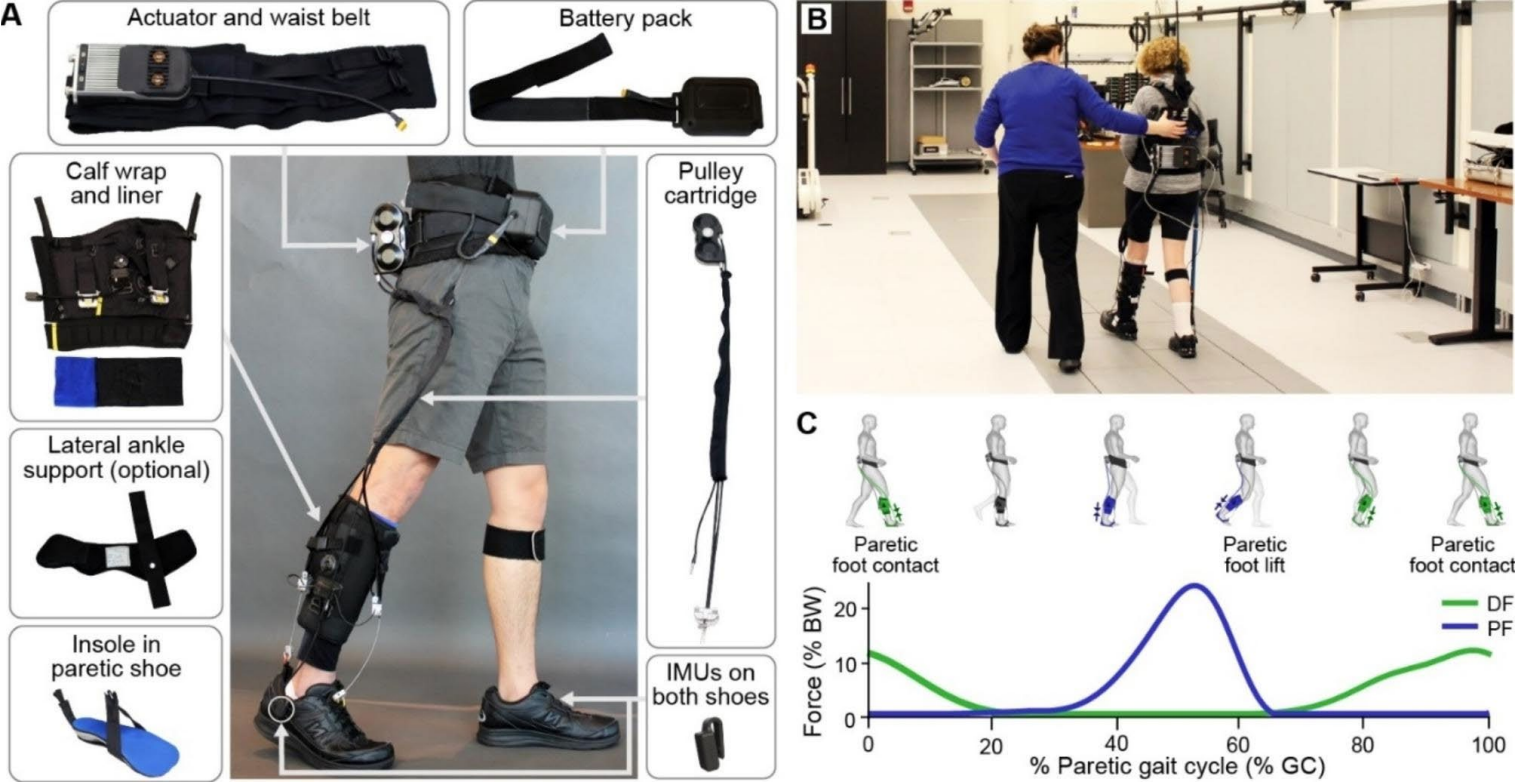
Overview of exosuit hardware and exosuit-generated assistance profile. (**A**) Components of a unilateral soft wearable robot (exosuit) designed to augment paretic ankle function during poststroke walking. The exosuit utilizes garment-like functional textile anchors and Bowden cables to generate assistive joint torques. Inertial measurement units (IMUs)attached to the shoe are used for online detection of gait events and system control. The system itself weighs 3.2 kg, with the different sizes of garments between 3.8 and 4.1 kg or between 3.6–8.9% (mean: 5.7%) of the subject’s body mass. Less than 14% of this mass was worn distally. (**B**) Study participant wearing the portable soft exosuit in the motion capture lab. (**C**) Example average exosuit forces from one participant. The exosuit generates plantarflexion (PF, blue) and dorsiflexion (DF, green) forces that are designed to restore the paretic limb’s contribution to forward propulsion during push-off and ground clearance, respectively

Motivated by these findings, we refined the form factor, usability, comfort, power consumption as well as the paretic gait event detection and cable position control algorithms of the portable exosuit, reducing its weight by nearly 25% to 3.8 kg (see *Methods*) [20]. The updated portable exosuit (Fig. 1) is designed to support long-distance overground walking, similar to everyday walking. Therefore, the purpose of this study is to extend our preliminary findings to a larger sample of individuals with chronic (> 6 months) poststroke hemiparesis by evaluating the immediate effects of plantarflexor and dorsiflexor assistance during continuous overground walking in the laboratory at a comfortable walking speed, compared to walking without an exosuit.

Our previous results indicate that baseline comfortable walking speed, a common clinical prognostic measure and predictor of intervention success, may impact the user’s ability to leverage exosuit assistance [19, 21, 23]. Therefore, the present study evaluates differences between people poststroke with comfortable walking speeds less than 0.93 m/s (limited community ambulators) and those with comfortable walking speeds greater than 0.93 m/s (full community ambulators), based on a recently introduced cut-off for functional stroke survivor groups [54]. Finally, the present study performs a thorough biomechanical evaluation, including effects on walking stability, control of foot landing, and slacking of the plantarflexor muscles. We hypothesize that the assistance provided by the updated portable exosuit will increase overground walking speed compared to walking without an exosuit by improving paretic ground clearance, foot landing, and propulsion. We expect these results to be enhanced in slower, more functionally limited participants. We further hypothesize that exosuit assistance will reduce the energy cost of transport, increase walking stability, and prevent plantarflexor muscle slacking.

## Methods

### Participants

Twenty individuals with hemiparetic stroke in the chronic phase of recovery were recruited for this study (9 F; age: 53 ± 11 year (mean ± standard deviation); chronicity: 8 ± 6y, Table 1), but one was excluded from analysis as they could not complete the continuous walking protocol. We recruited participants with a broad range of ambulatory functional levels based on clinical walking test outcomes from referring clinicians. Inclusion criteria included: aged between 18 and 80 years; diagnosis of stroke with gait deficiencies; and self-reported ability to walk independently with or without assistive devices continuously for at least 4 min. Exclusion criteria included severe aphasia, a speech or language disorder, serious co-morbidities, more than two self-reported falls in the previous month or limited ability to express needs and comprehend instructions (i.e., a score of 18 or lower on the Mini Mental State Exam (MMSE), or a score of 34 or lower on the Auditory Verbal Comprehension and Sequential Commands sections of the Western Aphasia Battery if the MMSE score was between 20 and 22). The protocol was approved by the Harvard Medical School Human Subjects Review Board, and medical clearance and signed informed consent were obtained for all participants.

**Table 1:** Participant characteristics

		Paretic side	Sex	Age (y)	Chronicity (y)	Type of stroke	Weight (kg)	Height (m)	Assistive device in session	FAC (0–5)	MAS (0–4)	Baseline speed (m/s)
Full ambulators	1	R	M	33	4.0	Hemorrhagic	86	1.81	None	5	0	0.97
	2	L	M	53	5.4	Ischemic	79	1.84	Arm sling	5	0	1.07
	3	R	F	32	10.3	Hemorrhagic	56	1.61	Lateral support	5	1+	1.03
	4	L	F	62	6.5	Ischemic	69	1.70	None	5	0	1.28
	5	R	F	48	6.5	Hemorrhagic	60	1.63	None	5	0	1.51
	6	R	F	56	8.8	Hemorrhagic	54	1.63	None	5	0	0.96
	7	L	M	59	9.0	Hemorrhagic	74	1.78	None	5	1	1.02
	8	L	M	46	12.7	Ischemic	81	1.75	None	5	0	0.96
	9	L	M	35	13.4	Ischemic	71	1.78	None	5	0	1.00
Limited ambulators	10	R	M	54	2.4	Hemorrhagic	81	1.76	Arm sling	4	0	0.53
	11	L	F	57	9.3	Ischemic	53	1.63	Cane & lateral support	4	0	0.45
	12	L	M	76	6.2	Hemorrhagic	99	1.82	None	5	0	0.88
	13	L	M	44	9.0	Ischemic	99	1.81	Cane & lateral support	4	1	0.70
	14	R	F	55	9.0	Hemorrhagic	114	1.65	Cane & lateral support	4	2	0.43
	15	L	F	49	4.3	Ischemic	43	1.60	None	5	1+	0.73
	16	L	M	62	4.3	Ischemic	65	1.75	Cane	5	1+	0.56
	17	L	M	57	3.5	Ischemic	111	1.69	Cane	5	0	0.69
	18	L	F	60	28.1	Ischemic	76	1.69	Cane & lateral support	4	1	0.67
	19	R	F	64	5.0	Ischemic	89	1.63	Cane & lateral support	4	2	0.34

FAC = functional ambulation category (score 0–5) rating overall gait function independence; MAS = modified Ashworth scale (score 0–4) describing muscle tone in the main plantarflexor muscles on the paretic ankle; baseline speed = baseline walking speed at the session

### Soft exosuit

The portable exosuit for paretic ankle assistance used in this study was first presented in [20]. The exosuit consists of a body-worn actuation unit mounted on a waist belt, a battery to generate mechanical power, Bowden cables to transmit mechanical power distally, a calf wrap to anchor the cable housings onto the shank, and a semi-rigid insole in the paretic shoe to anchor the cables to the shoe. Load cells (LSB200, Futek, Irvine, CA, USA) connected to the calf wrap measure forces applied by the cables, and two inertial sensors (MTi-3, XSens, Netherlands) mounted laterally on each shoe detect gait events relevant to control. All components together weigh between 3.8 and 4.1 kg depending on the garment sizes. An additional textile component to provide lateral support and prevent ankle inversion was added to the suit if necessary (n = 6, Table 1; Fig. 1).

The exosuit is improved from previous versions [19] in form factor, usability, comfort, and power consumption and includes new algorithms for gait event detection and force-based cable position trajectory generation (details in [20]). The actuation unit was designed to achieve motor torque and speed requirements calculated from experimental data while minimizing the weight and volume [20]. The actuation unit contains two motors (EC-4pole 22 90 W, Maxon Inc, USA) and a custom-made electronics board using an Atmel processor (SAME70N21, Atmel Co, USA) and motor drivers (Gold Twitter, Elmo Motion Control Ltd, Israel). Motor drivers were selected to be capable of 50 V and 60 A peak current, and a lithium iron phosphate battery was selected to supply 48 V with 1450 mAh capacity to allow for 90-minutes of continuous walking. The calf wrap was redesigned to eliminate the previous multi-articular structure of the exosuit and simplify donning and doffing.

The improved gait event detection algorithm used in this study does not rely anymore on identifying a heel strike event or foot flat phase, which are not always distinctly present in poststroke gait, but rather detects paretic and non-paretic toe-off and non-paretic mid-swing events from the inertial sensors [20]. These gait events segment the gait cycle into three phases that are relevant for the timing of the cable retraction and force generation: (1) paretic mid-stance, (2) paretic terminal stance, and (3) paretic swing to early stance.

The improved force-based cable position trajectory algorithm provides more consistent cable force during the more variable poststroke overground walking and reduced cable slacking (for technical details see [20]). The commanded cable position is updated each stride based on deviations from the desired peak force measured by the load cells [20]. The Bowden cable sheath for plantarflexion assistance connects the heel of the insole to the posterior side of the shank textile. Plantarflexion assistance was commanded to be 25% body weight (BW) but was set to 18% BW for one subject for comfort reasons. Plantarflexion cable force is generated at the end of the paretic mid-stance phase (at non-paretic mid-swing), reaches a commanded peak force during the paretic terminal stance phase, and diminishes before the paretic swing phase. Specifically, plantar flexion forces with a peak magnitude of 2.3 ± 0.04 N/kg or 23.9 ± 0.4% BW (average ± standard error) were generated during 30.5 ± 0.8 to 62.5 ± 0.7% of the gait cycle. These forces correspond to 28.1 ± 4.7% of the peak plantarflexion ankle moment when walking without the exosuit.

The Bowden cable sheath for dorsiflexion assistance connects the anterior side of the shank textile with the forefoot of the insole. The dorsiflexion cable force is generated at the beginning of the paretic swing phase (at paretic toe-off) and diminishes at the end of this phase, after paretic early stance (at non-paretic toe-off). The magnitude of dorsiflexion assistance is set based on visual observations by an experienced physical therapist to ensure sufficient ground clearance during swing and a smooth loading phase. The dorsiflexion cable generated tensile forces from 66.5 ± 0.7% to 25.0 ± 1.2% of the gait cycle. The peak magnitude of dorsiflexion force during swing was 1.6 ± 0.1 N/kg (16.0 ± 1.5% BW). Peak force magnitude during the limb loading phase was 2.6 ± 0.3 N/kg (26.3 ± 2.8% BW or 21.2 ± 24.1% of the peak dorsiflexion ankle moment over the loading phase when walking without an exosuit). Note that these descriptive values are based on 18 participants, as synced exosuit data was not available for one subject.

### Testing protocol

Prior to the testing session, participants completed a session in which the fit and comfort of the exosuit components were evaluated, and the required level of dorsiflexion assistance was visually determined (see Soft exosuit section). During the testing session, participants stood quietly for four minutes to assess their baseline energy expenditure. Participants walked continuously for two 5-minute conditions in the laboratory in random order: one baseline trial without wearing the exosuit, and one trial while wearing the active exosuit. Three participants with limited walking capacity walked for 4 min in both conditions. Participants walked on a 36.3 m indoor oval walking track at comfortable walking speed in both conditions, with their paretic side to the inside of the track (Supplemental Fig. 1). Before the start of the exosuit trial, participants walked for one to two laps with the exosuit active to verify comfort of the exosuit textile components and assistance magnitude, followed by a break. Participants were instructed to walk at their comfortable pace at the start of both conditions and no specific instructions on strategies in relation to exosuit-assisted walking were given, so to examine the natural response to the exosuit. There were at least 10 minutes of seated rest between conditions to reduce the effect of fatigue. Participants were supported via an overhead safety harness in case of a fall, and their vital signs were closely monitored throughout the testing session. No additional assistive devices were used during the testing, except a cane which was provided in both conditions if needed for safety (n = 7, Table 1). During each condition, three-dimensional gait analysis was performed to capture gait mechanics, energetics, and muscle activity.

### Measurement & analysis

#### Kinematics and kinetics

Full-body joint kinematics were measured by a motion-capture system (14 Oqus 7 cameras, Qualisys, Göteborg, Sweden; 200 Hz), for which 29 reflective markers and four 4-marker clusters (on the thighs and shanks) were placed on anatomical landmarks and segments. Eleven reflective markers were placed per leg on the greater trochanter, the medial and lateral epicondyles, the medial and lateral malleoli, medial and lateral side of the foot underneath the malleoli, the calcaneus, the second and fifth metatarsal heads and the front of the foot; as well as a sternum and six hip markers at the anterior superior iliac spines, iliac crest and a waist maker in between. To track the cables’ orientation necessary for determining exosuit-exerted moments, markers were also placed on the cable connection points at the shank and the foot. Ground reaction forces were collected by nine ground-embedded force plates (FP4060-10-2000 plates, Bertec, Columbus, OH, USA; 200 Hz). Load cells measured exosuit-exerted forces at 100 Hz.

Marker and ground reaction force data were low-pass filtered using a Butterworth filter with a 10 Hz cut-off frequency. Lower-body joint angles were calculated through inverse kinematics and total joint kinetics through inverse dynamics using motion analysis software (Visual3D, C-Motion, Rockville, MD, USA). Gait events were detected using a marker-based gait detection algorithm [24] and kinematic and kinetic data were time-normalized to 0-100% of the gait cycle (1001 time indices). Kinetic variables were normalized to body weight. Strides with full, single-foot landing on the force plates were selected for analysis, resulting in similar amounts of strides between exosuit conditions and between ambulator groups (limited community ambulators: 7.7 ± 3.9 [2–14] without exosuit and 10.2 ± 4.0 [4–16] with exosuit; full community ambulators: 11.4 ± 4.1 [5–18] without exosuit and 10.0 ± 2.5 [6–13] with exosuit.

### Biomechanical dorsiflexion targets: paretic ground clearance and foot landing

To assess the effect of dorsiflexion assistance during swing, minimal ground clearance was calculated as the difference in vertical distance of the marker placed on the fifth metatarsal head (lateral toe marker) between the minimum during swing (approximately mid-swing) and the average during mid-stance [25]. In addition, we examined the ankle dorsiflexion angle during mid-swing (when distance to the ground is minimal and ankle dorsiflexion is most critical) and compensatory hip hiking and circumduction. Hip hiking was defined as the maximum lateral difference in position of the center of gravity of the foot (from the model in Visual3D) during swing versus the vector from position at toe-off and at initial contact. Hip circumduction was defined as the maximum lateral difference in position of the center of gravity of the foot (from the model in Visual3D) during swing versus the vector from position at toe-off and at initial contact [21].

To assess changes in limb loading due to dorsiflexion exosuit assistance, we assessed foot placement during initial contact and the loading phase. A heel landing was defined as having a foot-to-floor angle (averaged over the first five samples of the gait cycle) of more than 10°. Additionally, ankle dorsiflexion angle at heel strike was reported. We assessed if the foot progressed to the ground in a controlled way through the smoothness of the ascent of the first peak of the vertical ground reaction force. The number of participants with a foot slap, defined as having a negative peak in the derivative of this force, was reported when walking with and without the exosuit.

### Biomechanical plantarflexion targets: paretic propulsion

Propulsion was evaluated as the average of the body center-of-mass (COM) propulsive power during the step-to-step transition (i.e., when the positive COM power crosses zero during the second double stance until toe-off). We previously demonstrated its correlation with changes in gait energetics during exosuit-assisted treadmill walking [23]. COM power was calculated as the dot product of the COM velocity vector estimated by the average velocity of the iliac crest markers and the individual limb ground reaction force vector [26]. To examine the effect of exosuit assistance on the ankle’s contribution to body propulsion, we evaluated the net (measured) paretic ankle moment and power. The peak values of the moment during push-off (from zero crossing in the propulsive phase to toe-off) and the average positive power during push-off were calculated.

EMG data from the gastrocnemius lateralis (GS) and soleus (SO) of both the paretic and non-paretic leg were measured using a wired system (Bagnoli, Delsys; 2000 Hz). EMG electrodes were carefully managed underneath the calf wrap to not disturb wires or signals, individually tested and remained attached for the duration of the testing session. EMG signals were band-pass filtered (4th order Butterworth, cut-off 20–450 Hz), rectified and low-pass filtered (4th order Butterworth, cut-off 10 Hz) to obtain a linear envelope. The same strides selected for kinematic and kinetic analysis were used for EMG analysis. Each muscle’s EMG was normalized to its maximum average muscle activity over the gait cycle during baseline walking without the exosuit, so the exosuit effect is clearly increasing (above 1) or decreasing (below 1) muscle activity relative to baseline. To evaluate changes in muscle activation between the two conditions, the area under the curve was calculated during the push-off phase (from non-paretic toe-off to paretic toe-off, to include plantarflexion muscle activation during mid-stance). The SO EMG data of one full community ambulator was excluded from analysis due to sensor issues.

### Clinical outcome: continuous comfortable walking speed

Average walking speed was derived from sternum marker data across the last two minutes of each trial to match the time window taken for the energy cost of transport.

### Secondary gait function outcomes: energy cost of transport and walking stability

To allow for steady-state assessment of metabolic cost, the last two minutes were used for calculation. Metabolic cost of transport was assessed by indirect calorimetry using a portable gas analysis system (K4b2, Cosmed, Roma, Italy). Metabolic power was calculated using a modified Brockway equation [27]. Net metabolic power was obtained by subtracting the metabolic power during the standing trial from the walking trials and was subsequently normalized by body weight and walking speed to yield net metabolic cost of transport. Metabolic cost data from two limited community ambulators were unavailable or had to be excluded from analysis due to malfunction in the portable pulmonary gas exchange measurement device.

Stability of walking was assessed by proxy metrics of step width (mediolateral distance between the center of gravity of the feet) and step length variability (coefficient of variance); both of which are related to different aspects of stability [28].

### Statistics

The first aim was to compare walking with the exosuit assistance versus without the exosuit across all participants, using either parametric paired 2-tailed t-tests or non-parametric Wilcoxon signed-rank tests. Variables were checked for a clear violation of the assumption of a normal distribution of the residuals through visual observation of QQ-plots supported by Shapiro-Wilk test results. To examine the contribution of the change in the main parameters (paretic ground clearance, foot landing and COM propulsion) to the change in continuous comfortable walking speed, a linear regression analysis was performed with an unconstrained intercept. Correlation of the variables to the dependent variable was checked, as well as normal distribution of residuals and absence of outliers as defined over three times the standard deviation.

The second aim was to evaluate the effect of the exosuit depending on the participant’s baseline functional ability. As several of the variables of interest are known to be related to an individual’s functional walking status [2, 29], for the second aim participants were divided into two main known functional groups, i.e. lower-level limited community ambulators (n = 10, with comfortable walking speed less than 0.93 m/s) and higher-level full community ambulators (n = 9, with speed above 0.93 m/s; see Table 1) [30]. We chose to use the most recent cut-off of 0.93 m/s [30], rather than the older accepted cut-off of 0.80 m/s [31, 32], to be in line with most recent literature. The exosuit effect was compared between ambulatory groups through the interaction effect in parametric 2-factor ANOVA tests (with/without exosuit as within-subject factor and limited vs. full ambulators as between-subject factor), or through non-parametric testing of the exosuit-induced percentage change between the two ambulatory groups using unpaired rank-sum tests. When an interaction effect was found, post-hoc analysis of exosuit-induced differences was performed within each ambulatory group using paired t-tests or Wilcoxon signed-rank tests. Linear regression analysis was performed to describe the contribution of the main variables to the change in walking speed per ambulatory group.

Average individual differences and standard errors are reported. For all tests, significance was set at p < 0.05. Statistical analysis was conducted in SPSS (v25, IBM Corp. Armonk, NY, USA).

## Results

Twenty individuals with chronic poststroke hemiparesis were recruited to participate in this study, but one individual was unable to complete the continuous walking protocol. We evaluated the effects of exosuit assistance on clinical and biomechanical parameters of continuous walking at a comfortable walking speed for 19 participants. A portable lightweight exosuit (Fig. 1) provided an average of 16.0 ± 1.5% body weight (BW) dorsiflexor assistance during swing to support ground clearance, 26.3 ± 2.8% BW dorsiflexor assistance during early stance to support limb loading, and 23.9 ± 0.4% BW plantarflexor assistance during push-off to support propulsion. Participants completed two trials of five minutes of continuous comfortable speed walking around an oval track, one without the exosuit and one with the exosuit active, in random order. The effects of walking with exosuit assistance were evaluated for all participants as well as between limited and full community ambulators. The data and statistical outcomes can be found in the Supplementary Data file.

### Propulsion

**Fig. 2:**
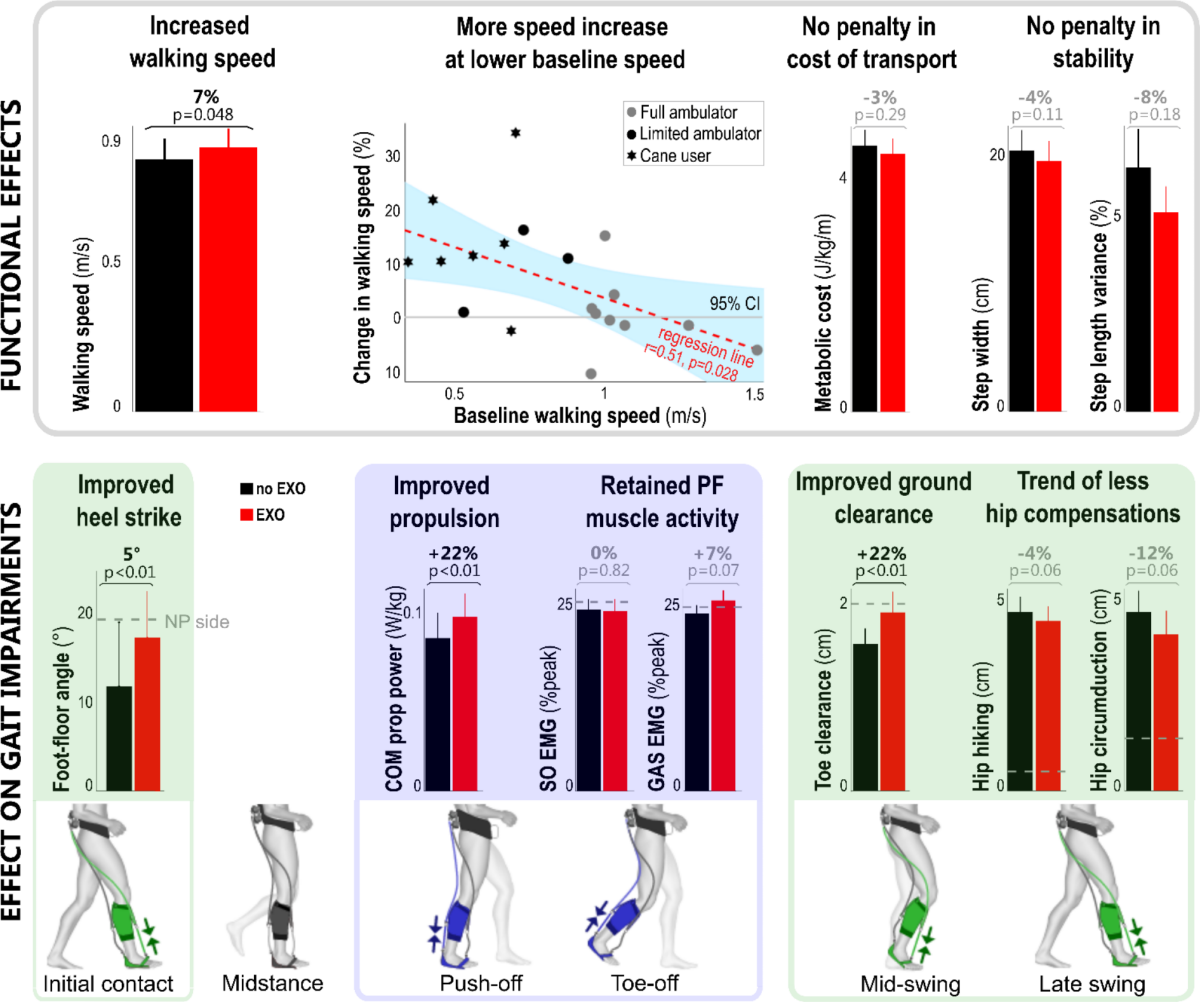
The average effect of exosuit assistance compared to baseline (without exosuit) walking across all poststroke participants (n = 19). Exosuit assistance improved walking speed, with lower baseline walking speeds resulting in higher speed increase, normalized flat foot landing to heel strike landing, increased propulsion power without reducing voluntary muscle activity during push-off, and improved ground clearance while reducing hip compensations. Bar graphs represent mean and standard error for walking with exosuit (red, EXO) and baseline walking without exosuit (black, noEXO) based on all participants (n = 19), except for the metabolic cost of transport (n = 17) and soleus muscle activity (n = 18). For some metrics, the non-paretic side is shown in a grey dashed bar for reference. The average exosuit effects across all participants are indicated in black (p < 0.05) or grey (p > 0.05). The Spearman r and p-value are reported for the correlation analysis on walking speed. With NP non-paretic side, P paretic side, SO Soleus muscle, GAS Gastrocnemius muscle, COM prop power the Center of Mass propulsive power, PF plantar flexion, CI confidence interval

**Fig. 3:**
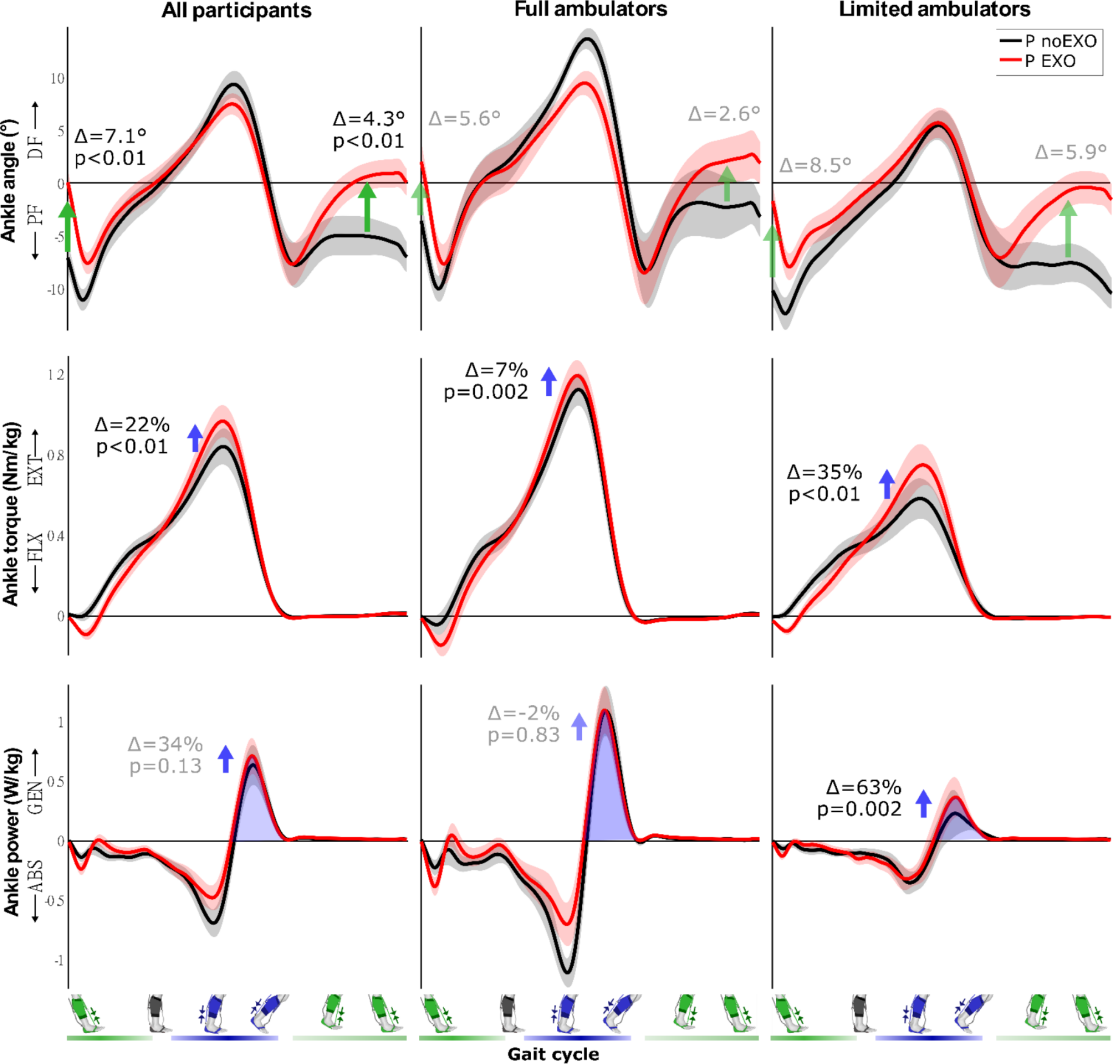
Effect of exosuit assistance on paretic ankle kinematics and kinetics. Time-normalized graphs of ankle angle, ankle moment and ankle power are shown, averaged over all stroke participants (n = 19, left column), full ambulators (speed > 0.93 m/s, n = 9, middle column) and limited ambulators (speed < 0.93 m/s, n = 10, right column). Walking without the exosuit is shown in black (noEXO), with the exosuit in red (EXO). Standard errors are indicated by the shaded area. Changes are indicated for ankle angle at initial contact, ankle angle mid swing, peak plantarflexion torque and positive power impulse during push-off, with non-significant average difference values between conditions in grey. Note that no interaction effect between exosuit and group was found for the ankle angle at initial contact or mid-swing; for the ankle torque and power the p-values from post-hoc testing are given for the full and limited community ambulators. The timing of dorsiflexion (green) and plantarflexion (blue) assistance are indicated at the bottom of the figure

**Fig. 4:**
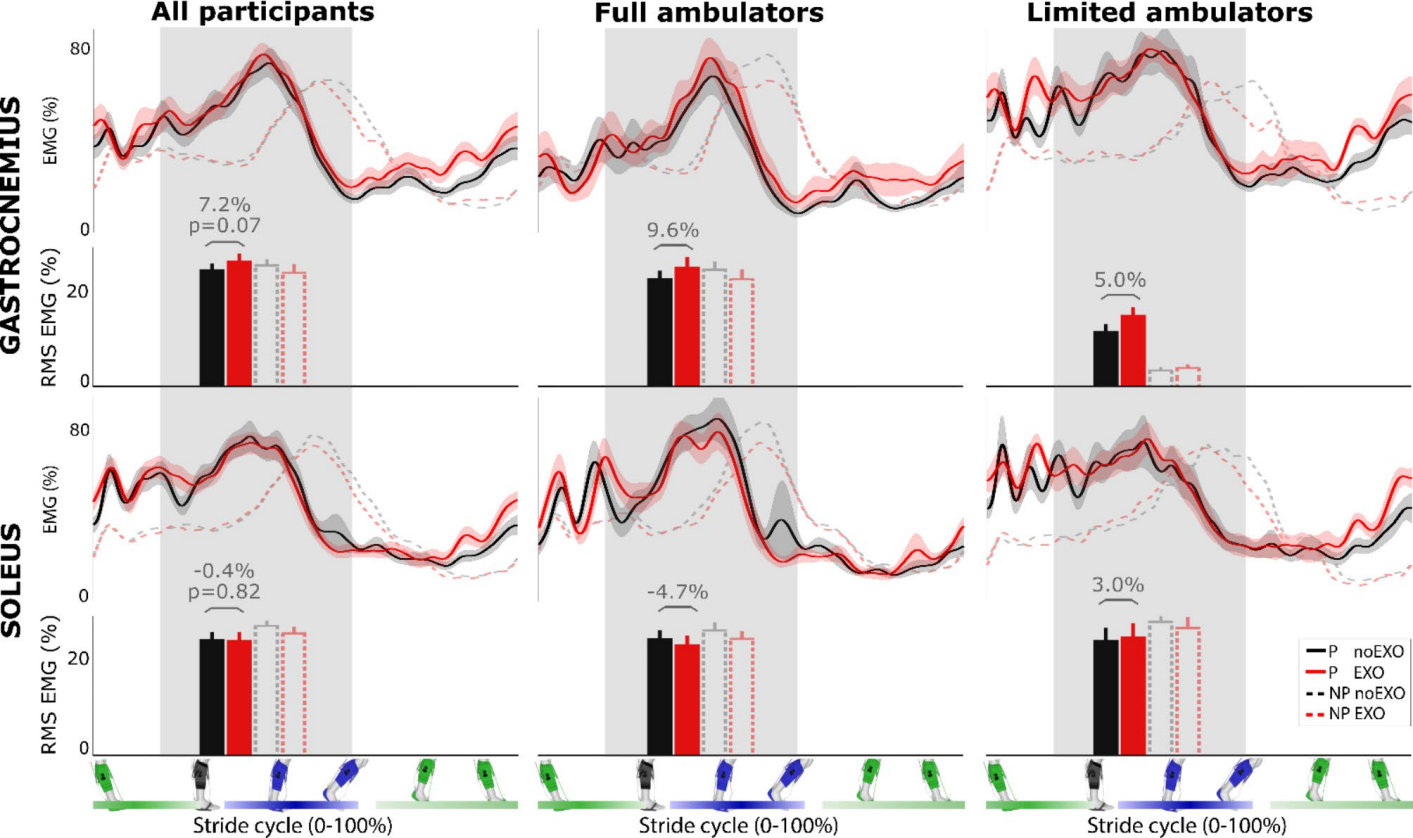
Effect of exosuit assistance on paretic lower leg propulsion muscle activation. Time-normalized graphs are shown averaged over all stroke participants (left column, n = 19 Gastrocnemius; n = 18 Soleus muscle), full ambulators (speed > 0.93 m/s, middle column; n = 9, Gastrocnemius and n = 8 Soleus muscle) and limited ambulators (speed < 0.93 m/s, n = 10, right column). Average and standard deviation (shaded area) are shown for walking without the exosuit (black, noEXO) and with exosuit assistance (red, EXO). Values for the non-paretic leg are given for context, time-normalized to the non-paretic leg initial contacts. Muscle activity was normalized to the maximum of that respective muscle during walking without exosuit per individual, for the paretic and non-paretic muscles separately. The group average root-mean-square (RMS) activity values and standard error are indicated for each muscle over the phase where the plantarflexor muscles are activated to achieve propulsion, from non-paretic toe-off to paretic toe-off (average time window indicated for the paretic muscles in grey, note that this time window is not indicated for the non-paretic muscles). The timing of dorsiflexion (green) and plantarflexion (blue) assistance are indicated at the bottom of the figure. No significant effects of exosuit assistance were found, and neither were any interaction effects between exosuit effect and group

As hypothesized, plantarflexor assistance augmented paretic propulsion — measured as the average positive center-of-mass (COM) power generated by the paretic limb during push-off — by 21.7 ± 5.4% (*P* = 0.003; Fig. 2) compared to walking without an exosuit. The ankle effort also changed: paretic peak plantarflexion moment showed a net increase of 21.6 ± 5.0% (*P* < 0.001), while changes in the average net paretic plantarflexion power during push-off were not observed (33.8 ± 11.2%, *P* = 0.125; Fig. 3). No changes were observed in the muscle activity of the paretic gastrocnemius (7.2 ± 3.6%, *P* = 0.073) and soleus (-0.4 ± 3.8%, *n* = 18, *P* = 0.818) averaged over the push-off phase (Fig. 4).

### Ground clearance and foot landing

As hypothesized, dorsiflexor assistance improved paretic ground clearance — measured as the minimum vertical distance between the toes and ground during swing — by 21.5 ± 6.6% (group average ± standard error; *P =* 0.002; Fig. 2) compared to walking without exosuit assistance. Ankle dorsiflexion angle at mid-swing increased by 4.3 ± 0.9° (*P* < 0.001; Fig. 3). There was a trend toward reduced paretic hip circumduction (-12.0 ± 7.6%, *P* = 0.060) and hip hiking (-3.5 ± 3.0%, *P* = 0.064) when walking with exosuit assistance.

As hypothesized, dorsiflexor assistance improved paretic foot placement at initial contact. Of the eight participants who demonstrated a flat foot landing, i.e., foot-to-floor angle smaller than 10° at initial contact, when walking without an exosuit, six of these established a heel landing when walking with exosuit assistance.

Averaged over all participants, the foot-to-floor landing increased by 5.4 ± 1.0° (*P* < 0.001; Fig. 2) and ankle dorsiflexion angle at initial contact increased by 7.1 ± 1.0° (*P* < 0.001; Fig. 3) with the exosuit assistance. Following heel landing, the foot progressed to the ground in a more controlled manner with exosuit assistance: the number of participants exhibiting foot slap (derived from the smoothness of the vertical ground reaction force) reduced from 11 without to six with exosuit assistance.

### Continuous walking speed

As hypothesized, comfortable walking speed increased on average by 6.7 ± 2.5% (0.04 ± 0.02 m/s; *P* = 0.048) when walking with exosuit assistance (Fig. 2). A regression model of the relationship between exosuit-induced changes in paretic ground clearance, foot landing and COM propulsion versus walking speed accounted for 82% of the variance in walking speed changes (*R*^*2*^ = 0.82, *F(3,15)* = 22.82, *P* < 0.001), with changes in foot landing (*β =* 0.362, *P =* 0.008) and propulsion (*β =* 0.747, *P <* 0.001) being independent contributors (ground clearance: *β =* 0.214, *P =* 0.089).

### Differential response between speed-based subgroups

Study participants were dichotomized into limited community ambulators (n = 10 slower) and full community ambulators (n = 9 faster) based on a baseline walking speed threshold of 0.93 m/s — a speed cutoff highly associated with the ability to walk more than 7,500 steps per day [30]. A Spearman correlation between the participants’ baseline and exosuit-induced change in walking speed confirmed the importance of baseline walking speed (r_s_=-0.51, p = 0.03). Between-group differences in the effect of walking with exosuit assistance were based on interaction effects found in a 2-factor (exosuit, subgroup) ANOVA.

**Fig. 5:**
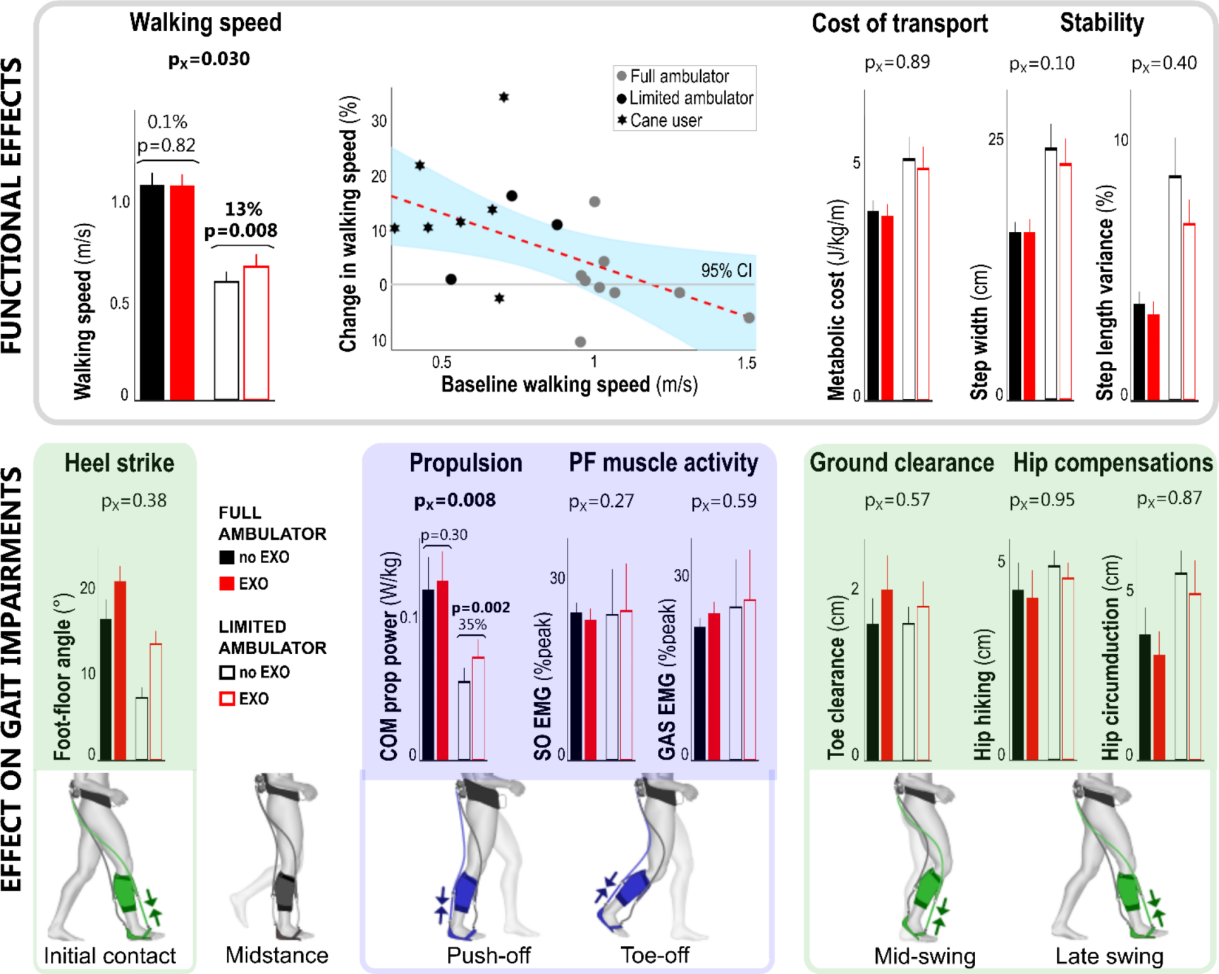
The effect of exosuit assistance compared to baseline walking in limited community ambulators versus full ambulator subgroups of poststroke participants. The interaction effect is given (p_X_-value), which indicates if there is a difference between the effect of exosuit assistance between the two groups. If this was found to be consistent, post-hoc t-tests (p-value) were performed (and indicated) per ambulatory group. Ambulatory groups were based on a cut-off walking speed of 0.93 m/s. Interaction effects were only found for walking speed and COM propulsion, as well as ankle moment and power during push-off (not shown). Bar graphs represent mean and standard error. With SO Soleus muscle, GAS Gastrocnemius muscle, COM prop power the Center of Mass propulsive power, PF plantar flexion

The effect of walking with exosuit assistance differed between subgroups for paretic COM propulsion (*P* = 0.008; Fig. 5) as well as the ankle contribution to propulsion with ankle moment (*P* = 0.002) and ankle power (*P* = 0.002). No differences were found for ground clearance (*P* = 0.565), heel landing (*P* = 0.380), or plantarflexor muscle activity (soleus: *P* = 0.274; gastrocnemius: *P* = 0.585). Limited community ambulators improved their propulsion with a 35.1 ± 7.0% increase in COM propulsion (*P* = 0.002), as well as a 34.9 ± 7.2% increase in paretic plantarflexor moment (*P* < 0.001) and 62.9 ± 15.0% in plantarflexor power (*P* = 0.002). Limited community ambulators increased their comfortable walking speed by 12.7 ± 3.2% (*P =* 0.008). In contrast, while full community ambulators increased their paretic plantarflexor moment with on average 6.8 ± 1.5% (*P* = 0.002), they did not change their COM propulsion (6.8 ± 5.1%, *P =* 0.301), paretic plantarflexor power (1.5 ± 7.9%, *P =* 0.833) or their walking speed (0.1 ± 2.4%, *P =* 0.820).

A regression model of the relationship between exosuit-induced changes in paretic ground clearance, foot landing and COM propulsion versus walking speed accounted for 76% of the variance in walking speed changes (*R*^*2*^ = 0.76, *F(3,6)* = 6.33, *P* = 0.03), with changes in propulsion (*β =* 0.829, *P =* 0.007) being an independent contributor (foot landing: *β =* 0.369, *P =* 0.13; ground clearance: *β =* 0.126, *P =* 0.57).

### Energy cost of transport and walking stability

Contrary to our hypothesis, we did not observe changes in the energy cost of transport – measured as weight-normalized energy expenditure per distance walked (-2.6 ± 2.78%, n = 17; P = 0.288). There were also no changes in walking stability – represented by step width (-3.9 ± 2.7%, *P* = 0.107) and step length variability (-7.6 ± 9.2%, *P* = 0.175). Differences between speed-based subgroups were not observed for the energy cost of transport (*P* = 0.888), step width (*P* = 0.101), or step length variability (*P* = 0.404).

## Discussion

This study evaluated the effect of walking with a portable, lightweight soft robotic exosuit during continuous overground walking in people poststroke in a single testing session. Specifically, it contrasts the effect of exosuit assistance in limited and full community ambulators. We investigated a wide range of biomechanical metrics, including those directly related to the impairments targeted with the exosuit’s dorsiflexion assistance (ground clearance and foot landing) and plantarflexion assistance (COM propulsion, ankle mechanics, voluntary muscle activation). We also included clinically relevant gait metrics such as continuous walking speed, metabolic cost of transport and stability, which bear important relevance to patients’ quality of life and effectiveness of gait rehabilitation [8, 33].

### Increased continuous walking speed

Walking with exosuit assistance increased walking speed on average by 6.7% (0.04 m/s) over all participants compared to walking without an exosuit. Limited community ambulators – those who walked at baseline speeds slower than 0.93 m/s – increased walking speed by 13% (0.08 m/s). This is an important demonstration of an ankle-assisting exosuit considerably increasing overground walking speed in more limited individuals poststroke, for whom improving mobility is critical for their social participation [8]. Furthermore, while limited community ambulators often use canes to improve mobility and increase walking speed [34–36], our data demonstrated that exosuits can complement cane-assisted walking with six out of seven cane users increasing their walking speed (17%; Fig. 2).

Full community ambulators did not increase their walking speed on average, possibly due to less capacity for improvement. They also received less push-off assistance relative to their baseline ankle moment (7% vs. 35% for limited community ambulators). Interestingly, in our previous smaller sample of individuals poststroke (0.67–1.11 m/s; 3 full community and 3 high-level limited community ambulators) we did find an increase in comfortable walking speed with exosuit assistance, and although these comparisons were made across different days, this underlines that a subset of full community ambulators may also benefit from assistance [22]. This study examined immediate, in-session gait improvements induced by exosuit assistance alone, and participants may further benefit when given longer familiarization time, guidance on interaction with the exosuit, or cues to target gait impairments. As the required regimen for optimal familiarization to robotic devices is largely unknown in poststroke participants, this remains a future research topic.

### Augmented propulsion and preserved propulsive muscle activity

As forward propulsion is a key determinant of walking function, improving paretic propulsion is often the main goal of poststroke gait training [9, 37]. By delivering relatively small amounts of assistance to the paretic ankle (28% of peak baseline ankle moment), the exosuit improved forward propulsive body COM power by 22% averaged over all participants during overground walking, similar to the 23% we previously found for treadmill walking [23]. Building on the positive relation between ankle push-off power and COM propulsive power we found previously [23], we now provide the first evidence that paretic ankle function improvements translate to increased walking speed overground.

Even though patient participation is vital to effective poststroke gait training, the effect of active assistive devices on muscle activity is not often measured. Previous studies have reported reductions in muscle activity in both small groups of poststroke [38] and able-bodied individuals [39–43] using active ankle assistive devices. Interestingly, we did not find a reduction in paretic plantarflexion muscle activity during push-off with exosuit support, with gastrocnemius muscle activity even demonstrating a trend of an increase in activity. Using ultrasound and tendon tensiometry may be promising paths forward to study in more detail the effect of exosuit assistance on paretic plantarflexion muscle dynamics throughout the gait cycle [44, 45].

Consistent with walking speed, the effect of exosuit assistance on propulsion was also dependent on the ambulatory group. Limited community ambulators leveraged the assistance to increase their ankle power and walking speed, while full community ambulators maintained their speed. This aligns with our previous finding of the negative correlation between baseline walking speed and improvement in propulsion during treadmill walking with an exosuit [19]. Optimal onset timing of plantarflexion assistance differed between individuals in previous work [19], so individualizing the trajectory of the delivered exosuit force to adapt to the user’s needs may increase propulsion and walking speed in full community ambulators. Optimization through human-in-the-loop and deep learning algorithms have shown great potential to tailor wearable technology [46–49], with further improvements anticipated through tailoring direct measures of neuromuscular function [50, 51], but challenges related to for instance restrictions in walking duration and user experience have to be met for successful application to rehabilitation settings [52].

### Restored ground clearance without restricting push-off

Reduced ground clearance during swing is a common poststroke gait deficit related to instability and falls [2, 4, 7], and was presently shown to improve by 22% with exosuit assistance. There are several strategies to achieve ground clearance, including dorsiflexing the ankle, flexing the knee and/or compensating at the hip. Dorsiflexion assistance directly targeted and increased ankle dorsiflexion by 4°, which is almost five times the minimal detectable change [53] and comparable to a 5° change we found during treadmill walking [19–21]. Increases of 10° dorsiflexion have been reported using other active ankle devices for people poststroke; however, these studies included subjects with pronounced ground clearance deficits [54–58]. As a result of the improved ankle dorsiflexion, hip compensatory behavior showed a trend of reducing. The trend of reducing hip hiking was less pronounced compared to our previous treadmill study (5% vs. 27%), but the current reduction in hip circumduction was larger (13% vs. 2%), possibly due to sample differences in baseline hip compensations and habitual strategies underlying overground speed changes [21]. Next to improving ankle dorsiflexion and reducing hip compensations, plantarflexion assistance may also have the potential to increase knee flexion during swing in a group of stiff-knee patients because of increasing push-off power and restoring a more normal leg trajectory.

### Improved foot placement

The control of foot placement at initial contact is often impaired in individuals poststroke, leading to a flat foot landing and/or foot slapping when rotating to the ground. This landing pattern has been suggested to impede weight transfer to the paretic limb [5, 59]. While several assistive devices have been designed to address these foot landing deficits poststroke, few studies have directly evaluated their impact. The dorsiflexion assistance provided by the exosuit during the early loading phase was designed to encourage a heel landing followed by a controlled progression to a flat foot. On average, the exosuit supported a paretic heel landing at initial contact by increasing foot-to-floor angle by 5°, through increasing the ankle dorsiflexion angle at initial contact by 7° which is over four times the 1.6° minimal detectable change of the latter variable [53]. Only two of 19 participants maintained their flat foot landing with exosuit assistance, and the occurrence of foot slap disappeared in nearly half of the participants with this presentation. Tuning the dorsiflexion assistance during swing and loading response separately may further improve foot placement and ground clearance in those individuals with foot landing deficits.

### Preserved gait energetics and stability

Walking with exosuit assistance did not increase the metabolic cost of transport, which is in line with our previous proof-of-concept overground results [22] and other studies applying assistive ankle devices to poststroke gait [38, 60]. This result indicates that factors expected to worsen the cost of transport, such as the added weight of the system and exosuit-induced increase in walking speed, were at minimum compensated for by the exosuit assistance and subsequent reduction in costly hip compensatory behavior. The average difference in cost of transport between walking with and without the exosuit assistance was − 2.6%. For this calculation, we had missing metabolic cost data from two limited community ambulators that were among the top five best responders to the exosuit assistance in terms of walking speed. Interestingly, there was a large variability in the exosuit-related change in cost of transport (from − 24% to + 14%). We hypothesized that the push-off assistance, improved foot placement and reduced need for costly hip compensation would contribute to reductions in the cost of transport, which we did not find. Despite the lack of group level change in the metabolic cost of transport with exosuit assistance, this could be considered a positive finding for future use of exosuits in gait training as it doesn’t add significant burden and preserves the intensity of training. If achieving a reduction in the metabolic cost of transport is a goal (i.e., for assistive orthotic applications), allowing individuals more adaptation time [38] or applying higher levels of assistance [61] may be required.

Despite fall risk being a concern in people poststroke, the effect of assistance applied by portable robots on gait stability has, to our best knowledge, not yet been assessed. While traditional exoskeletons for gait training provide body weight support and inherent stability, the soft character of exosuits likely require users to actively control their stability. Our proxy measures of stability - step width and step length variability - indicated that exosuit assistance did not destabilize participants, even within the limited community ambulatory group. Further studies on the effect of exosuit assistance on the control of dynamic gait stability, particularly after sufficient familiarization time, would be insightful.

### Study limitations

Some limitations should be noted. First, we chose to dichotomize our data based on walking speed, as it is a common clinical prognostic measure and an indicator of the success of an intervention. We used the most recent cut-off (0.93 m/s) in the field of poststroke gait [30]. This specific cut-off did not influence our subgroup analysis, as repeated analysis using an older but previously widely recognized cut-off of 0.80 m/s [31, 32] yielded the same results. Creating functional subgroups does not allow for identifying continuous predicting variables, and larger follow-up studies are needed to identify the strongest predictors for responders to exosuit assistance. Second, most participants who required a cane for safe overground ambulation were in the limited ambulators and we cannot exclude that leaning on the cane altered the effectiveness of the exosuit assistance in these individuals and thus this ambulatory group. To reduce the effect on the study, both conditions were performed with the cane. Third, this study did not include a condition with the exosuit worn but not active, as a previous study has already shown the negligible effect of the additional weight on energetics [22], nor a condition with only dorsiflexion support to disentangle the different support types on typical poststroke impairments [62]. Finally, while this study did evaluate longer continuous walking, it was measured during level, undisturbed walking. Future research should establish the effectiveness of the exosuit during more complex and challenging walking tasks, preferably in the community [51, 63].

### Outlook: potential for poststroke gait training

Although improving walking ability is one of the major goals of poststroke rehabilitation, current gait training does not provide sufficient therapeutic gain, which is a major motivation for the development and study of rehabilitation robotics [64]. One of the compelling aspects of a portable exosuit is its potential to provide assistance during both treadmill and overground gait training, and this study demonstrated the translation of our previously reported gait improvements from treadmill to overground walking in a larger, more heterogeneous group of poststroke individuals. The lightweight, portable design of the exosuit potentially supports three fundamental aspects of poststroke gait training: dosage, specificity, and intensity [33]. The immediate restorative benefits of the exosuit presented here underline this potential. First, through increased paretic push-off and walking speed, the exosuit may facilitate taking more steps during training, and thus a higher dose. Second, the exosuit could facilitate training specificity by targeting ankle impairments and hip compensatory strategies thus allowing patients to focus on specific aspects of their gait. Third, preserved muscle activity and metabolic cost of transport may facilitate training intensity. Moreover, the exosuit provides a promising platform for gait training innovation, as assistance can be tuned to an individual’s specific gait impairments and adapted within a training session. These benefits are confirmed with increasing evidence of the benefits of using an exosuit during gait training [65–67], including exploiting the exosuit paradigm to apply resistance to increase plantarflexor effort [68]. While the current study reveals immediate improvements in walking ability without any instructions on how to leverage the exosuit assistance, finding effective cues may further increase and accelerate improvement in poststroke walking ability [69].

## Conclusions

This study shows that walking with a portable, lightweight soft robotic exosuit improved overground walking speed and quality in a large group of people poststroke within a single testing session. As designed, the dorsiflexion assistance improved ground clearance during swing and foot landing during initial contact, and the plantarflexion assistance improved propulsion and decreased hip compensations. These improvements were larger in the group of limited ambulators, underlining the importance of individualized assistance. The assistance did not negatively affect the walking stability, energy cost of transport, or voluntary muscle activation of the calf muscles during push-off, which are important for the effectiveness of gait rehabilitation. The immediate restorative benefits of the exosuit presented here underline its promise for rehabilitative gait training in poststroke individuals.

### Electronic supplementary material

Below is the link to the electronic supplementary material.

Additional File Table 1: Main Group Effects

**Additional File Figure 1**: Overview of the measurement set-up. (**A**) Study participant walking with the portable soft exosuit, while motion capture cameras and floor-mounted force plates capture the movement. (**B**) In addition to the exosuit, participants wore an indirect calorimetry system, EMG electrodes and reflective markers. Safety measures included guarding by a licensed therapist, a safety harness connected to an overhead rail and the use of a cane on the nonparetic side if needed. (**C**) Study participants walked continuously for five minutes on an overground track of 36.3 m in length

## Data Availability

The data that support the findings of this study are included in this published article and its supplementary information files. The underlying individual-level outcomes are available from the corresponding authors on reasonable request.
