# Supplementary figures and images for: Effects of a soft robotic exosuit on the quality and speed of overground walking depends on walking ability after stroke
Source: J Neuroeng Rehabil. 2023 Sep 1;20:113. doi: 10.1186/s12984-023-01231-7 (PMC10474762; doi:10.1186/s12984-023-01231-7)

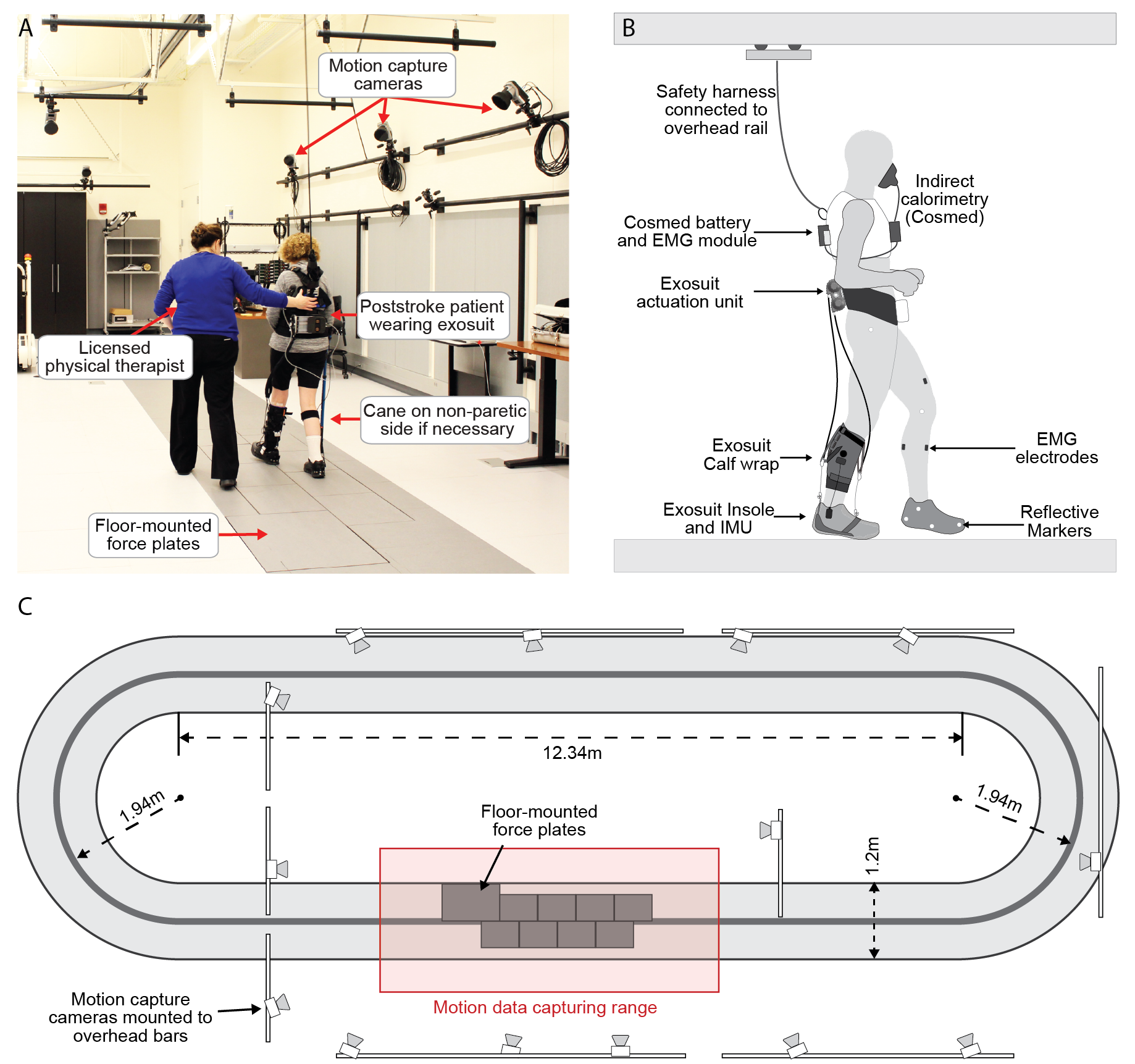

Supplement: Supplementary file 2 — Additional File Figure 1: Overview of the measurement set-up. (A) Study participant walking with the portable soft exosuit, while motion capture cameras and floor-mounted force plates capture the movement. (B) In addition to the exosuit, participants wore an indirect calorimetry system, EMG electrodes and reflective markers. Safety measures included guarding by a licensed therapist, a safety harness connected to an overhead rail and the use of a cane on the nonparetic side if needed. (C) Study participants walked continuously for five minutes on an overground track of 36.3 m in length [file 12984_2023_1231_MOESM2_ESM.png]
